# Quantifying Serum Level of Glycochenodeoxycholic Acid Using High Performance Liquid Chromatography in Obstructive Jaundice Patients

**DOI:** 10.1155/2013/508368

**Published:** 2012-11-05

**Authors:** Toar J. M. Lalisang, Metta Sinta Sari Wiria, Vivian Sutikno, Melva Louisa, Arie Estuningtyas

**Affiliations:** ^1^Digestive Surgery Division, Cipto Mangunkusumo Hospital and Faculty of Medicine, University of Indonesia, Jakarta 10430, Indonesia; ^2^Department of Surgery, Faculty of Medicine, University of Indonesia, Medical Staff Building 4th Floor, Jalan Diponegoro No. 71, Jakarta 10430, Indonesia; ^3^Department of Pharmacology & Therapeutic, Faculty of Medicine, University of Indonesia, Jakarta 10430, Indonesia

## Abstract

*Introduction.* Accumulation of glycochenodeoxycholic acid (GCDC) in serum has a clinical significance as an inductor of pathological hepatocyte apoptosis, which impairs liver function. Inhibition of GCDC accumulation can be used as a marker in therapy. This study was aimed to quantify the serum level of GCDC in obstructive jaundice patients. *Methodology.* GCDC acid level in the serum was quantified using high performance liquid chromatography (HPLC) technique according to Muraca and Ghoos modified method. It was performed before and after decompression at day 7 and day 14. The sample was extracted with solid phase extraction (SPE) technique on SPE column. The results were analyzed using SPSS V 16.0 (*P* < 0.05) and quantified with standard curve on GCDC acid. *Result.* There were 21 cases with range of GCDC acid serum level before decompression was 90.9 (SD 205.5) *μ*mol/L and day 7 after decompression decreased to 4.0 (SD 46.4) *μ*mol/L and then increased to 11.3 (SD 21.9) *μ*mol/L (*P* < 0.05). This method could separate GCDC acid on serum with good resolution, high precision and accuracy, and linear calibration curve on measured level range. *Conclusion*. HPLC can quantify GCDC acid serum on obstructive jaundice patients and can be used to support its pharmacokinetic study.

## 1. Introduction

The accumulation of glycochenodeoxycholic (GCDC) acid in obstructive jaundice patients is cytotoxic, can harm hepatocytes, and impairs liver function [1, 2]. GCDC acid is a primary biliary acid directly divided from cholesterol in hepatocytes and conjugated with amino acid glycyne or taurin and becomes a conjugate of glycine or taurin [3, 4]. GCDC acid, as a dose dependent inductor of apoptosis in hepatocyte and kinase protein C, can be used as a parameter of pathological apoptosis of hepatocyte and impaired liver function [5].

Several methods can be used to measure biliary acid level in the body fluid (serum or biliary fluid). Enzyme immunoassay method, which is mostly used to measure the total level of biliary acid, cannot be applied to measure primary, secondary, and tertiary biliary acid distinctively [6]. Liquid or gas chromatography is the chosen method for quantifying level of biliary acid [1]. Several animal studies have shown that high performance liquid chromatography (HPLC) can measure GCDC acid serum faster and precisely [6].

This study measures the GCDC acid serum in severe obstructive jaundice patients through biliary tract decompression models, using Muraca and Ghoos modified methods.

## 2. Methodology

This study was performed on obstructive jaundice patients, mostly due to periampullary tumor, with serum bilirubin above 10 mg/dL, who were admitted in the period from December 2007 to January 2009. Patients with active hepatitis A, B, or C, liver malignancy, severe liver impairment with massive ascites, past experience of decompression procedure, and chronic liver failure were excluded. We used conventional open cholecystectomy as the bile decompression model to provide changes of GCDC acid level.

Principle for quantifying conjugates of primary biliary acid (glycochenodeoxycholic, taurochenodeoxycholic, glycodeoxycholic, taurodeoxycholic acid), in the sample serum extracted by solid phase extraction (SPE) using Sepak C-18 column, with methanol solution after being mixed with the base NaOH. The extract was concentrated using the N_2_ gas evaporation method, then set by high performance liquid chromatography using analytical column C18 with eluated mixture of methanol-phosphate buffer solution pH 4.5 (75 : 25). The result of eluated separation is detected using ultra violet beam with the wave length of 205 nm. The chromatogram result was then recorded in the computer using the Empower software. GCDC acid level was set using the calibration curve, as a regression line between the chromatographed areas versus standard level of GCDC acid. High performance liquid chromatography tool is made using Waters Alliance 2695 Separation Module system, Waters 2487 dual wave length, absorbance detector, with auto sampler, degasser and Empower software. Separation of sample was done in an Atlantis C_18_ 5 *μ*m 3.9 × 150 mm Analytical column, protected by Sentry Guard Symmetry C_18_ 5 *μ*m 3.9 × 20 mm Guard column, using one set of vacuum filtration from Millipore, SPE extractor from JT Baker, evaporization tool of N_2_ gas with water bath 40°C.

Test sample: *Spike* sample is GCDC acid standard on model serum and serum sample from cholestasis patients.

Chemical material test: sodium GCDC (Sigma-Aldrich Pte.Ltd, Product No. G0759 1 G); glycodeoxycholic acid (Sigma-Aldrich Pte.Ltd, Product No. 9910 1 G); methanol (E Merck, HPLC grade), acetone; KH_2_PO_4_, and H_3_PO_4_ (E Merck); membrane filter (Whatman, pore size 0.45 *μ*m); Sepak SPE column, C-18, (Waters Associate, Cat. no. WAT 049010), and bond elute LRC-C18 (Varian, SPE column).

## 3. Setting Procedure

### 3.1. Validation Of Setting Method

(1) Set the LOD and LOQ of GCDC acid standard solution.
(a) Produce the primary standard solution (std.P) and working standard solution (std.W) glycochenodeoxycholic acid and glycodeoxycholic acid on methanol.
  (b) Produce the primary standard solution (std.P) and the working standard solution (std.W) GCDC acid on the serum (spike sample-1).
    (b.1) Make the std.P-GCDC acid solution on the serum, measure carefully 200 *μ*L of std.P—the GCDC acid solution on methanol, add the model serum up to 2 mL (level 200 *μ*g/mL).
    (b.2) Make the std.W-GCDC acid solution on the serum, measure carefully 200 *μ*L of std.P-GCDC acid solution on the serum (1.3), then add the model serum up to level: 1; 5; 10; 25; 50; 100 *μ*g/mL.
  (c) Extraction procedure with solid phase extraction (SPE).
    (c.1) To 1.0 mL of serum (sample or model) add 0.5 mL of GCDC solution (standard) and 7.5 mL mixed solution of mobile phase solution—0.2 M NaOH (6 : 8, v/v), then mix it until homogenous.
    (c.2) Add the sample to the SPE C-18 column that already activated with flowing methanol and water, respectively. Then rinse the sample with flowing 10.0 mL of water, 3.0 mL of acetone solution 10% on the water, and 10.0 mL of water, respectively. The solution speed is 10 mL/min.
    (c.3) The sample then extracted with 2.0 mL methanol. The eluat result then evaporized with N_2_ gas flow on 37°C.
    (c.4) The residual then dissolved back to 0.5 mL mobile phase with vortex mixer for 30 seconds, then 10 *μ*L of that solution is injected to HPLC.
  (d) Setting by the high performance liquid chromatography (HPLC).
   The chromatography system: the analytical column use the *Atlantis *C_18_ 5 *μ*m 3.9 × 150 mm, protected by *Sentry Guard Symmetry *C_18_ 5 *μ*m 3.9 × 20 mm *Guardcolumn*. The mobile phase consisted of a mixed solution methanol—a phosphate buffer solution contained 2.5 KH_2_PO_4_ and 20 mM NaOH with pH 4.5, regulated by H_3_PO_4_ 85% (75 : 25). Flow speed: 1.0 mL/min, and detected by ultra violeton the wave length of 205 nm. Width area of the chromatogram is integrated electronically (*Empower software*) (Figure 2).
  (e) The LOD estimation based on the issued level of S/N >3, and the LOQ estimation based on the issued level of S/N >10.
(2) Calibration curve.
 Calibration curve made of the spike samples (on serum) with range level: 0.5; 1.0; 5.0; 10.0; 25.0; 50.0; 100.0 *μ*g/mL. Set the linearity of calibration curve/regression line.
(3) Set the precision (coefficient of variation) and accuracy of the setting method.

### 3.2. *In Vivo* Implementation

1. The extraction sample test 1.2 (serum) is suited with the extraction procedure used in the setting of the validation method (point A no. 2).
2. The sample test level with HPLC according to the procedure was set using the validation setting (point A no. 3).
3. The calibration curve was set in each sample test setting.

## 4. Result and Discussion

This study, which was performed on patients with characteristics mentioned in Table 2, succeeded in quantifying the level of GCDC and glycodeoxycholic (GDC) acid in the serum of severe obstructive jaundice patients using the modified methods from Muraca and Ghoos. The chromatography method that is mostly used is high performance liquid chromatography (HPLC) with ultra violet (UV) detector or spectromass.

**Figure 1:**
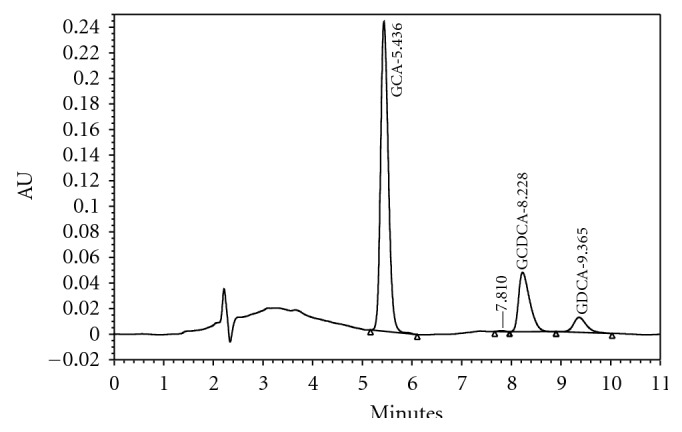
The high performance liquid chromatography chromatogram data on the comparing agents with Rf scores. (1) GCDC acid (8.23) (2) glycodeoxycholic acid (9.37).

**Figure 2:**
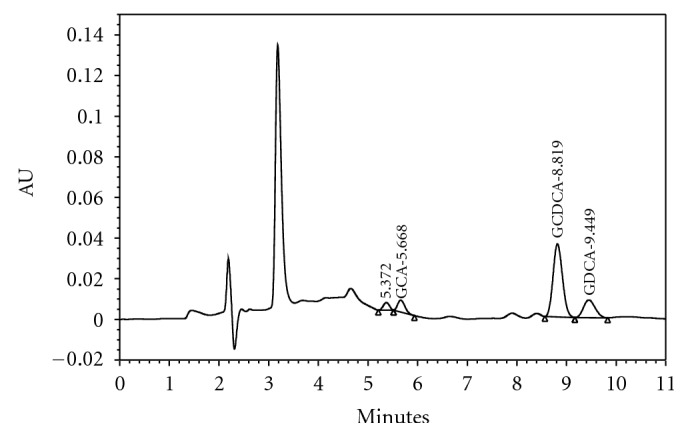
The high performance liquid chromatography chromatogram from the sample extract ofbile obstruction patients serum contained GCDC acid (GCDCA), with glycodeoxycholic acid (GDCA) as the inner standard.

Because the GCDC acid is a very polar molecule, the extraction or isolation of this molecule needs to use solid-liquid extraction technique. The modification was done on the SPE column to acquire the desired sensitivity. The modification was also done on the chromatography system, which is an alternative to the degree of acidity on the mobile phase to 4.5 and using a more sensitive analytical homogenous column (C18). With these modifications, the GCDC acid and glycodeoxycholic acid separation resulted in a better resolution and a sharp chromatogram peak. This modification method can be used to quantify the GCDC acid and glycodeoxycholic acid serum (Figure 1).

The glycodeoxycholic acid is a secondary bile acid used as an internal standard because it can be detected and gives a better chromatogram peak. With the chromatography system, the pessimistic hypothesis that glycodeoxycholic acid on the sample can decrease the accuracy of level estimation can be solved by decreasing the extracted estimated sample without using the glycodeoxycholic acid as the internal standard.

The precision and accuracy set on the level 1.0; 10.0; 50.0 *μ*g/mL for intraday validation gives score 3.39; 6.27; 3.05 and 3.61; 6.50; 3.23, and for interday validation gives score 4.74; 10.47; 11.36 and 6.94; 7.62; 10.48, that fulfill the validation requirement (Table 1). The calibration curve on range level 0.5; 1.0; 5.0; 10.0; 25.0; 50.0; 100.0 *μ*g/mL gives score *r* ^2^ = 0,9997. The implementation of the modified method on *in vivo* samples (cholestasis patients) gives a better result which is the level can be quantified using the obtained calibration curve (Table 2).

This study quantifies the serial levels of GCDC acid serum in severe obstructive jaundice patients, which proves that there is an accumulation of glycochenodeoxycholate in the circulation of obstructive jaundice patients.

Several studies reported that the accumulation of bile acid can harm the liver via apoptosis and necrosis and can cause kidney failure, diarrhea, sepsis, arrhythmia, epistaxis, andasthma-like disease [7–9]. Although bile acid can cause several diseases, its serum level is still unfrequently included in clinical practices or in hospitals as a liver function test or hepatocelular damage test [10]. 

**Figure 3:**
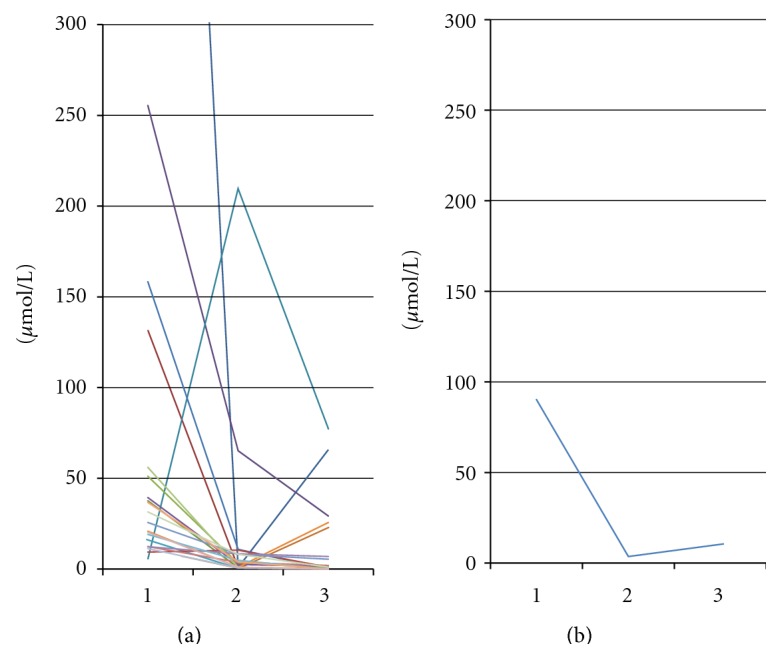
(a) Profileand (b) rate level of glycochenodeoxycholate (1) before decompression [90.9 (SD 205.5)  *μ*mol/L], (2) day 7 after decompression [4.0 (SD 46.4) *μ*mol/L] and (3) day 14 after decompression [11.3 (SD 21.9) *μ*mol/L] (*P* < 0.05).

Accumulation of GCDC acid existed on each case of severe obstructive jaundice and was followed by pathological apoptosis of hepatocytes. This finding suits the animal study result [11]. The aim of this study was to measure the difference between level of glycochenodeoxycholate in the pre- and postdecompression process of the bile duct. The decompression process significantly decreased level of glycochenodeoxycholate as an apoptosis inductor of hepatocyte from 90.9 (SD 205.5) *μ*mol/L to 4.0 (SD 46.4) *μ*mol/L and then increased to 11.3 (SD 21.9) *μ*mol/L (*P* < 0.05) (Figure 3). Decreased accumulation of glycochenodeoxycholate in the postdecompression stage is due to the regurgitation stop and reuptake process of glycochenodeoxycholate by hepatocytes and then it is excreted to canaliculi. Increasing of hepatocyte apoptosis and accumulation of glycochenodeoxycholate in obstructive jaundice patients showed the role of glycochenodeoxycholate as an inductor of hepatocyte apoptosis via extrinsic apoptosis pathway, and this finding suits with the animal study result [5, 12]. However, in postdecompression stage there was a significant decrease in hepatocyte apoptosis and glycochenodeoxycholate level, so it can be concluded that a decrease on glycochenodeoxycholate level through bile duct decompression mechanism is an inhibitor of pathologic hepatocyte apoptosis.

The differences from the previous study are the glycochenodeoxycholate level and hepatocyte apoptosis, which are not dose dependent in this study [2, 5]. This was due to difference in model and study design. Induction of apoptosis by glycochenodeoxycholate in the previous study was usually found in the hepatocyte culture, and on the other hand, this study showed that glycochenodeoxycholate was found on the liver tissue. The glycochenodeoxycholate level in this study is 30 times higher than normal level (according to Douglas, the glycochenodeoxycholate level in nonjaundice patient is between 0.2 and 3.2 *μ*mol/L) [13]. After decompression, the level of glycochenodeoxycholate on 12 cases was normal, mean while on the other 9 cases, the level decreased to 4 times lower than normal range.

A few recent studies were looking for glycochenodeoxycholate antagonist such as fructose, calpain, PMA, and brefeld in A as apoptosis inhibitors [2, 5, 11]. In this study, the antagonist form of glycochenodeoxycholate as inhibitor of pathologic hepatocyte apoptosis was reached through the decompression stage of bile duct.

## 5. Conclusion

From this study we can summarize that the modified analysis method can be used to quantify level of GCDC acid on serum samples and furthermore the method can be used to support the GCDC acid pharmacokinetic study.

## Figures and Tables

**Table 1:** Data on precision and accuracy of glycochenodeoxycholate acid on human serum on the prevalidation study.

Intraday validation						
Nominal concentration		1.0		10.0		50.0
Precision (%)		3.39		6.27		3.05
Accuracy (%)		3.61		6.50		3.23

Interday validation						
Nominal concentration		1.0		10.0		50.0
Precision (%)		4.74		10.47		11.36
Accuracy (%)		6.94		7.62		10.48

**Table 2:** Baseline characteristics of patients included in the study.

Characteristics	
Sex—female/male	7/14
Age	44 (12.7)
Diagnosis	
Periampullar malignancy	15
Periampullar benign tumor	6
Routine laboratory examination (before decompression)	
Hb (g/dL)	11.2
Ht (%)	32.7
WBC (1000/*μ*L)	11.3
Platelets (1000/*μ*L)	260
Ureum/Creatin (mg/dL)	35.5/0.9
Blood glucose (mg/dL)	104

## References

[1] Muraca M., Choos Y. (1985). Glyco-7*α*,12*α*-dihydroxy-5*β*-cholanic acid as internal standard for high-pressure liquid chromatographic analysis of conjugated bile acids. *Journal of Lipid Research*.

[2] Spivey J. R., Bronk S. F., Gores G. J. (1993). Glycochenodeoxycholate-induced lethal hepatocellular injury in rat hepatocytes. Role of ATP depletion and cytosolic free calcium. *Journal of Clinical Investigation*.

[3] Eichhorst S. T. (2005). Modulation of apoptosis as a target for liver disease. *Expert Opinion on Therapeutic Targets*.

[4] Schattenberg J. M., Galle P. R., Schuchmann M. (2006). Apoptosis in liver disease. *Liver International*.

[5] Gonzalez B., Fisher C., Rosser B. G. (2000). Glycochenodeoxycholic acid (GCDC) induced hepatocyte apoptosis is associated with early modulation of intracellular PKC activity. *Molecular and Cellular Biochemistry*.

[6] Jones M., Chen H., Ouyang W. (2003). Method for bile acid determination by high performance liquid chromatography. *Journal of Medical Sciences*.

[7] Emerick M., Deodhar J., Windle M., Cuffari C. (2006). Cholestasis. *eMedicine Specialties Pedeatic Gastroenterology*.

[8] Fogarty B. J., Parks R. W., Rowlands B. J., Diamond T. (1995). Renal dysfunction in obstructive jaundice. *British Journal of Surgery*.

[9] Padillo J., Puente J., Gómez M. (2001). Improved cardiac function in patients with obstructive jaundice after internal biliary drainage: hemodynamic and hormonal assessment. *Annals of Surgery*.

[10] Heaton K. W. (1979). Bile salt tests in clinical practice. *British Medical Journal*.

[11] Sodeman T., Bronk S. F., Roberts P. J., Miyoshi H., Gores G. J. (2000). Bile salts mediate hepatocyte apoptosis by increasing cell surface trafficking of Fas. *American Journal of Physiology*.

[12] Malhi H., Gores G. J., Lemasters J. J. (2006). Apoptosis and necrosis in the liver: a tale of two deaths?. *Hepatology*.

[13] Douglas J. G., Beckett G. J., Nimmo I. A. (1981). Clinical value of bile salt tests in anicteric liver disease. *Gut*.

